# Real-world effectiveness of antipsychotic medication in relapse prevention after cannabis-induced psychosis

**DOI:** 10.1192/bjp.2025.72

**Published:** 2026-04

**Authors:** Antti Mustonen, Heidi Taipale, Alexander Denissoff, Venla Ellilä, Marta Di Forti, Antti Tanskanen, Ellenor Mittendorfer-Rutz, Jari Tiihonen, Solja Niemelä

**Affiliations:** Faculty of Medicine and Health Technology, Tampere University, Tampere, Finland; Department of Clinical Neuroscience, Karolinska Institute, Stockholm, Sweden; Department of Psychiatry, Seinäjoki Central Hospital, Seinäjoki, Finland; Department of Forensic Psychiatry, Niuvanniemi Hospital, University of Eastern Finland, Kuopio, Finland; School of Pharmacy, University of Eastern Finland, Kuopio, Finland; Department of Psychiatry, Faculty of Medicine, University of Turku, Turku, Finland; Addiction Psychiatry Unit, Department of Psychiatry, Turku University Hospital, Wellbeing County of South-West Finland, Turku, Finland; Social, Genetic and Developmental Psychiatry Centre, Institute of Psychiatry, Psychology and Neuroscience, King’s College London, London, UK; South London and Maudsley NHS Mental Health Foundation Trust, London, UK; National Institute for Health Research (NIHR) Mental Health Biomedical Research Centre at South London and Maudsley NHS Foundation Trust and King’s College London, London, UK; Neuroscience Center, University of Helsinki, Helsinki, Finland; Center for Psychiatry Research, Stockholm Region, Stockholm, Sweden

**Keywords:** Cannabis, cannabis-induced psychosis, relapse, antipsychotics, psychotic disorders

## Abstract

**Background:**

Cannabis use is linked to treatment non-adherence and relapses in psychotic disorders. Antipsychotic medication is effective for relapse prevention in primary psychoses, but its effectiveness after cannabis-induced psychosis (CIP) remains unclear.

**Aims:**

To examine the effectiveness of antipsychotic medication for relapse prevention following the first clinically diagnosed CIP.

**Method:**

A cohort of 1772 patients (84.1% men) with incident CIP was identified from the Swedish National Patient and Micro Data for Analyses of Social Insurance registers. The primary outcome was hospitalisation due to any psychotic episode. Drug use data were collected from the Prescribed Drug Register and modelled into drug use periods using the PRE2DUP method. A within-individual Cox regression model was used to study the risk of outcomes during the use of different oral or long-acting injectable (LAI) antipsychotics compared with non-use.

**Results:**

The mean age at first diagnosis was 26.6 years (s.d. = 8.3). Of the cohort, 1343 (75.8%) used antipsychotics and 914 (51.3%) experienced psychosis hospitalisation during the follow-up. Any antipsychotic use was associated with a decreased risk of psychosis hospitalisation (adjusted hazard ratio (aHR) 0.75; 95% CI 0.67–0.84). Specific antipsychotics associated with decreased risk included aripiprazole LAI (aHR 0.27; 95% CI 0.14–0.51), olanzapine LAI (aHR 0.28; 95% CI 0.15–0.53), clozapine (aHR 0.55; 95% CI 0.34–0.90), oral aripiprazole (aHR 0.64; 95% CI 0.45–0.91), antipsychotic polytherapy (aHR 0.74; 95% CI 0.63–0.87) and oral olanzapine (aHR 0.81; 95% CI 0.69–0.94).

**Conclusions:**

In particular, LAIs, clozapine and oral aripiprazole were associated with a decreased risk of psychosis relapse following CIP. Prescribers should consider using more LAIs for better treatment outcomes after CIP.

The association of cannabis use and primary psychoses such as schizophrenia is well established, with studies suggesting early onset and heavy cannabis use associating with risk of psychotic outcomes.^1^ In people with established psychotic disorder, cannabis use disorder (CUD) is common, ranging from 21% in schizophrenia to 36% in first-episode psychosis (FEP) samples,^2^ and most patients with psychotic disorders continue use after remission of a psychotic episode.^3^ Cannabis use worsens prognosis of psychotic disorders and associates with increased non-adherence to antipsychotic medications,^4,5^ risk of relapse, more intense and frequent in-patient treatment^6–9^ and treatment resistance.^10^

Compared to primary psychotic disorders, prognosis of substance-induced psychoses (SIPs) is much more unclear. According to a recent meta-analysis by Murrie et al,^11^ among SIPs, cannabis-induced psychosis (CIP) associates with worst prognosis in terms of schizophrenia conversion with one out of three later converting into schizophrenia. This manifests especially among young males and those with familial predisposition to psychosis.^12,13^ This matter is topical as recent studies report rising rates of CIP during the past ten years,^14,15^ which may be a proxy for increased burden of disease from psychotic disorders associated with cannabis use. This might be preventable with treatment optimisation.

Treatment of psychotic disorders impacted by cannabis use

Psychosis relapses associate with poor prognosis, and antipsychotic medication is effective in preventing relapses in primary psychoses.^16^ Although effectiveness of antipsychotic medication in patients with psychotic disorder and CUD have been studied in clinical trials^17–19^ and community samples,^20–22^ only one study has focused on long-term outcomes.^20^ Recently, using nationwide Swedish register data, we reported that use of any antipsychotic medication was associated with 33% risk reduction of psychotic relapse in people with first clinically diagnosed psychosis and comorbid CUD. In our study, use of clozapine and long-acting injectable (LAI) formulations of antipsychotic medications were associated with lowest risk of psychotic relapse.^20^

However, data on effectiveness of antipsychotic medication after CIP is scarce, and data is based on a few small clinical trials^23,24^ and case reports.^25^ Most importantly, there are no reports where effectiveness of antipsychotic medications is studied in CIP populations on any long-term outcome. Further, our current diagnostic guidelines consider SIP as a brief psychotic syndrome that occurs during or following psychoactive substance use and no guidelines on how antipsychotic medications should be prescribed exist. Thus, it remains unclear how pharmacotherapy should be optimised after CIP.

Using data from linkage of nationwide registers in Sweden, our aim was to examine whether antipsychotic medication is effective in preventing admissions to hospital caused by psychosis relapse after first onset of clinically diagnosed CIP (*n* = 1772). Secondary outcomes included hospital admission caused by substance use disorder (SUD) and any somatic disorder.

## Method

### Study population

Data are based on several Swedish nationwide registers that include all individuals with Swedish residency. All Swedish residents are assigned a unique personal identification number, which enables linkage between various registers after de-identification. These registers include the National Patient Register (NPR), Micro Data for Analyses of Social Insurance (MiDAS) register, Cause of Death Register (CDR), Prescribed Drug Register (PDR) and Longitudinal Integration Database for Health Insurance (LISA) register.

The NPR includes data on in-patient and specialised out-patient care periods, while the MiDAS register includes data on sickness absence and disability pension, namely data on periods during which individuals have received sickness benefits because of health-related incapacity for work. From the NPR and MiDAS register we sampled all individuals aged 16–64 years that were registered for the first time with clinically diagnosed CIP (ICD-10^26^ code F12.5) between January 2006 and December 2021. They were identified based on not having a previous (from 1997 to 2005) episode of SIP (F1x.5) or schizophrenia-spectrum disorder (F20–F29) or bipolar disorder (F30 and F31) to account for solely incident psychosis cases. Sociodemographic data (age, gender, educational level, country of birth, occupational data) were derived from the LISA register.

We used data from the REWHARD consortium that was supported by the Swedish Research Council (grant number 2021-00154). The research project was approved by the Regional Ethics Board of Stockholm, Karolinska Institutet, Stockholm, Sweden (decision 2007/762-31 and Dnr 2021-06441-02). According to current Swedish law, the use of registry data for research purposes does not require informed consent from individuals held in these registries.

### Exposure variables

Medication data were gathered from the PDR from July 2005 to December 2023 and were categorised into antipsychotics based on Anatomical Therapeutic Chemical (ATC) classification^27^ code N05A, excluding N05AN01 (lithium). Antipsychotics were categorised into oral and LAI formulations. Most common antipsychotics were second-generation oral and LAI antipsychotics, namely risperidone and paliperidone LAI, oral and LAI aripiprazole, oral and LAI olanzapine, clozapine and quetiapine. Use of other antipsychotic medications, ‘other oral antipsychotic monotherapy’, use of two or more concurrent antipsychotics, ‘antipsychotic polytherapy’, and all LAI formulations of first-generation antipsychotics, ‘first-generation LAI’ (FG-LAI), were pooled to provide adequate power for analysis. Antipsychotic medication use was compared with non-use of antipsychotics. Medication data were modelled into medication use periods (i.e. when medication use started and ended) with the PRE2DUP (from prescription drug purchases to drug use periods) method.^28^ Exposure to antipsychotics was modelled in a time-dependent manner and updated in the models when any change to antipsychotics in use happened (i.e. switch, add-on, discontinuation). Medication data with less than five events are not reported.

### Outcomes

The primary outcome was hospital admission caused by any psychotic episode, that is, primary psychotic disorder (ICD-10 codes F20–F29) or any SIP (ICD-10 codes F1x.5 as a main diagnosis). Secondary outcomes were (a) hospital admission caused by SUD (ICD-10 codes F10–F19 as a main diagnosis) and (b) hospital admission caused by any somatic disorder (ICD-10 codes A00–N99, excluding F00–F99 as a main diagnosis). Hospital admission caused by any somatic disorder was included as a marker of serious somatic problems leading to hospital admission, as antipsychotics can have adverse effects.

### Covariates

Temporal order of antipsychotic medication treatments, time since cohort entry and time-varying use of other psychotropic medication were adjusted in analyses. These medications were categorised based on their ATC classification as medications for SUDs (N07BB, N07BC), medications for attention-deficit hyperactivity disorder (N06BA), mood stabilisers (N03AF01, N03AG01, N03AX09, N05AN01), antidepressants (N06A), benzodiazepines and related drugs (N05BA, N05CD, N05CF).

### Statistical methods

We used a within-individual design with a stratified Cox regression model in which each individual formed his or her own stratum. This reduces selection bias^29^ as it controls for time-invariant factors such as genetics and baseline comorbidities. Among the incident CIP sample, we calculated adjusted hazard ratios (aHRs) and 95% confidence intervals comparing the risk of outcomes during time periods of use of specific antipsychotics with time periods of non-use of antipsychotics. The follow-up time was reset to zero after the outcome event, meaning that main outcomes were treated as recurrent events. Patients were followed up from CIP – diagnosis until emigration (LISA register), death (CDR) or end of the data linkage (December 2023), whichever occurred first. As a sensitivity analysis, between-individual Cox regression analyses were conducted for the main outcome (see the Supplementary Material online available at https://doi.org/10.1192/bjp.2025.72). Statistical significance was considered at >0.05. Statistical analyses were performed using SAS version 9.4 for Windows (SAS Institute Inc., Cary, NC, USA; https://www.sas.com/fi_fi/software/iml-sas9.html). Forest plot figures were created using R version 4.1.1 for Windows (R Foundation for Statistical Computing, Vienna, Austria; https://www.R-project.org/127).

## Results

The sample totalled 1772 individuals with CIP, of which 1490 (84.1%) were men and the mean (s.d.) age was 26.6 (8.3) years at first diagnosis. Most had low or medium educational level (Table 1). A total of 995 patients (56.2%) had work income at baseline, 96 (5.4%) had more than 90 days of sickness absence during the calendar year before study entry and 123 (6.9%) were receiving a disability pension at study entry.

**Table 1:** Characteristics of the incident cannabis-induced psychosis sample (*n* = 1772)

Variable	Frequency	Percent
Age (years)		
16–19	232	13.09%
20–24	681	38.43%
25–29	399	22.52%
≥30	460	25.96%
Gender		
Female	282	15.91%
Male	1490	84.09%
Born in Sweden		
No	501	28.27%
Yes	1271	71.73%
Education^a^		
Elementary	830	46.83%
High school	752	42.44%
University/college	190	10.72%
Income from work		
No	777	43.85%
Yes	995	56.15%
Sickness absence previous year		
No	1495	84.37%
1–90 days	181	10.21%
≥90 days	96	5.42%
Disability pension at cohort entry		
No	1649	93.06%
Yes	123	6.94%

a. Elementary (9 years or less), high school (10–12 years) and university/college (>12 years of education).

The individuals were followed up at 8.26 years (s.d. 4.35) on average. Of the sample, 1343 (75.8%) used antipsychotics during the follow-up. Most commonly used antipsychotics were oral olanzapine (1013; 57.2% of the sample), antipsychotic polytherapy (675; 38.1%), quetiapine (385; 21.7%), oral aripiprazole (331; 18.7%) and oral risperidone (261; 14.7%). For the primary outcome, medication-specific results by the number of individuals prescribed medications, number of events, person years and aHR are shown in Table S1 in the Supplementary Material.

During the follow-up, 69.7% (*n* = 1235) were re-diagnosed with any cannabis use-related diagnosis (F12.x), and 52.0% (*n* = 921) specifically with CIP (F12.5), 27.9% (*n* = 495) with F12.1 Harmful use of cannabis and 27.5% (*n* = 488) with F12.2 Cannabis dependence.

### Risk of hospital admission caused by any psychotic episode

**Fig. 1:**
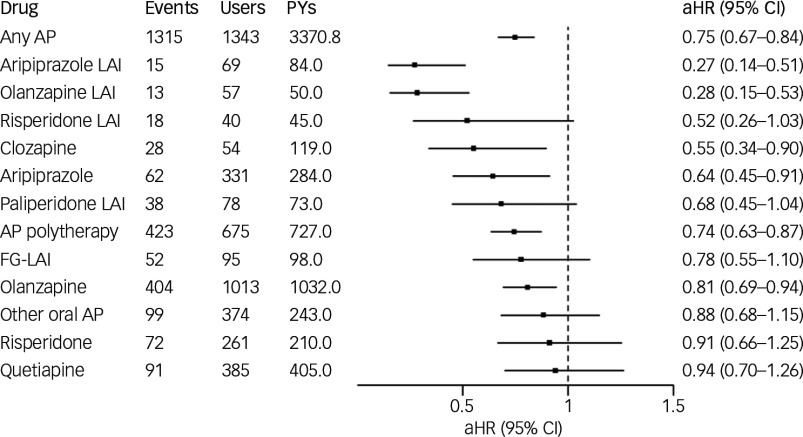
Association of antipsychotic (AP) use with hospital admission caused by psychotic relapse. FG-LAI, first-generation antipsychotic long-acting injectable; LAI, long-acting injectable; aHR, adjusted hazard ratio; PYs, person years.

During the follow-up, 914 (51.3%) individuals experienced hospital admission caused by psychosis. In total, there were 3920 hospital admissions, of which 57.2% were caused by primary psychotic disorder and the rest caused by substance-induced psychotic disorder. The most common specific categories within this outcome were F29 (unspecified non-organic psychosis; 23.5% of all hospital admissions), F12.5 (CIP; 23.0% of all hospital admissions) and F20 (schizophrenia; 17.9% of all hospital admissions). Any antipsychotic use (versus no-use) was associated with decreased risk of hospital admission caused by any psychotic episode (aHR 0.75; 95% CI 0.67–0.84). Of the specific antipsychotics, aripiprazole LAI (aHR 0.27; 95% CI 0.14–0.51), olanzapine LAI (aHR 0.28; 95% CI 0.15–0.53), clozapine (aHR 0.55; 95% CI 0.34–0.90), oral aripiprazole (aHR 0.64; 95% CI 0.45–0.91), antipsychotic polytherapy (aHR 0.74; 95% CI 0.63–0.87) and oral olanzapine (aHR 0.81; 95% CI 0.69–0.94) were associated with decreased risk, whereas risperidone LAI (aHR 0.52; 95% CI 0.26–1.03), paliperidone LAI (aHR 0.68; 95% CI 0.45–1.04), FG-LAIs (aHR 0.78; 95% CI 0.55–1.10), other oral antipsychotic monotherapy (aHR 0.88; 95% CI 0.68–1.15), oral risperidone (aHR 0.91; 95% CI 0.66–1.25) and quetiapine (aHR 0.94; 95% CI 0.70–1.26) did not reach statistical significance (Fig. 1).

### Risk of hospital admission caused by SUD

**Fig. 2:**
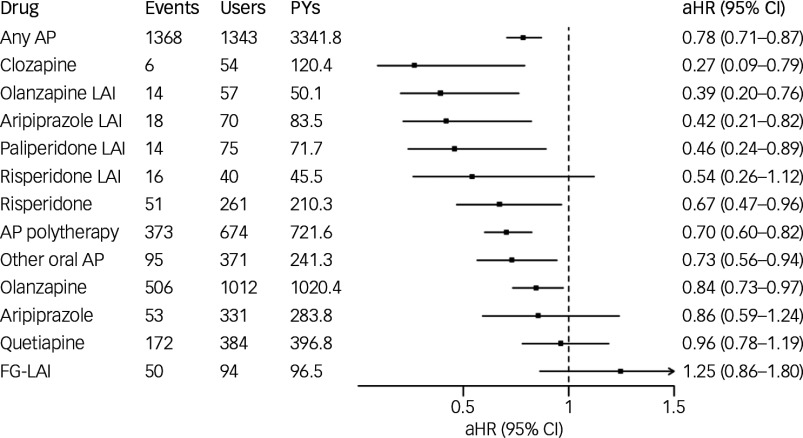
Association of antipsychotic (AP) use with hospital admission caused by substance use disorder. FG-LAI, first-generation antipsychotic long-acting injectable; LAI, long-acting injectable; aHR, adjusted hazard ratio; PYs, person years.

During the follow-up, 1021 (57.6%) experienced hospital admission caused by SUD. Any antipsychotic use (versus no-use) was associated with decreased risk of hospital admission caused by SUD (aHR 0.78; 95% CI 0.71–0.87). Of the specific antipsychotics, clozapine (aHR 0.27; 95% CI 0.09–0.79), olanzapine LAI (aHR 0.39; 95% CI 0.20–0.76), aripiprazole LAI (aHR 0.42; 95% CI 0.21–0.82), paliperidone LAI (aHR 0.46; 95% CI 0.24–0.89), oral risperidone (aHR 0.67; 95% CI 0.47–0.96), antipsychotic polytherapy (aHR 0.70; 95% CI 0.60–0.82), other oral antipsychotic monotherapy (aHR 0.73; 95% CI 0.56–0.94) and oral olanzapine (aHR 0.84; 95% CI 0.73–0.97) were associated with decreased risk, whereas risperidone LAI (aHR 0.54; 95% CI 0.26–1.12), oral aripiprazole (aHR 0.86; 95% CI 0.59–1.24), quetiapine (aHR 0.96; 95% CI 0.78–1.19) and FG-LAIs (aHR 1.25; 95% CI 0.86–1.80) did not reach statistical significance (Fig. 2).

### Risk of hospital admission caused by somatic disorder

**Fig. 3:**
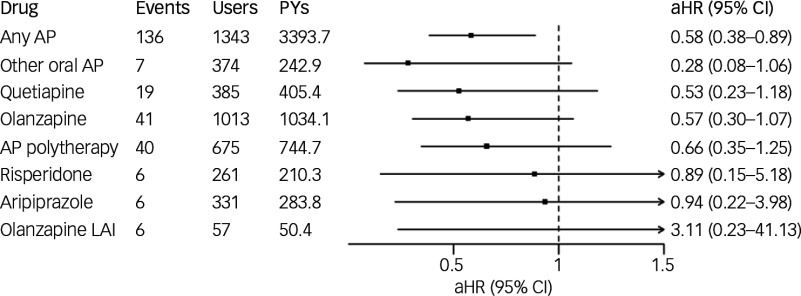
Association of antipsychotic (AP) use with hospital admission caused by somatic disorder. LAI, long-acting injectable; aHR, adjusted hazard ratio; PYs, person years.

During the follow-up, 306 (17.2%) experienced hospital admission caused by somatic disorder. By ICD-10 main categories, the most common reasons for somatic hospital admissions were diseases of the digestive system (K) at 20.6%, diseases of the musculoskeletal system and connective tissue (M) at 18.4%, diseases of the nervous system (G) at 12.6%, diseases of the respiratory system (J) at 10.4% and diseases of the circulatory system (I) at 9.9%. Any antipsychotic use (versus no-use) was associated with decreased risk of hospital admission caused by somatic disorder (aHR 0.58; 95% CI 0.38–0.89). Most specific antipsychotics lacked statistical power for drug-level analysis, and none were associated with either an increased or decreased risk (Fig. 3). There were too few events (less than five) to run medication modelling for aripiprazole LAI, paliperidone LAI, risperidone LAI, FG-LAIs and clozapine.

## Discussion

Using nationwide Swedish register data, we report that antipsychotic medications, especially LAIs, were effective in preventing hospital admission caused by psychosis relapse and SUD after first diagnosis of CIP. While a recent expert consensus statement recommends the use of LAIs in FEP,^30^ our findings suggest this to be beneficial even in CIP. This is especially important as cannabis use is associated with non-adherence to medication in psychotic disorders.^9^

LAI formulations of aripiprazole and olanzapine were associated with the lowest risk of psychosis relapses after CIP, followed by clozapine, risperidone LAI, oral aripiprazole, antipsychotic polytherapy and oral olanzapine. Paliperidone LAI, risperidone (neither oral nor LAI), FG-LAI, other oral antipsychotic monotherapy or quetiapine did not reach statistical significance for effectiveness. Compared to non-use, the use of aripiprazole and olanzapine LAIs was associated with a 72–73% reduction in the risk of psychosis relapse. In contrast, their oral counterparts were associated with a 19–36% risk reduction. Although neither the oral nor LAI formulation of risperidone reached statistical significance for effectiveness in preventing psychosis relapses, these medications followed a similar pattern (LAI 48% *v.* oral 9%). This pattern was also observed in our sensitivity analyses using a between-individual design. These results suggest that LAIs are more effective than oral antipsychotics (excluding clozapine) in preventing psychosis relapse after CIP, thereby encouraging the use of LAIs for better treatment outcomes.

Antipsychotics other than strong dopamine antagonists performed similarly in relapse prevention after CIP than after first onset of schizophrenia with or without SUD^16,31^ or FEP and CUD.^20^ The findings are plausible in the context of a similar relapse rate in SIP versus FEP^32,33^ and similar rates of individuals converting into schizophrenia after CIP versus FEP.^11^ In contrast to these previous studies, none of the oral or LAI strong dopamine antagonists associated with a statically significant decreased risk of psychosis relapse. Possible reasons for some of these results are the low number of users and non-adherence to the oral formulations of these antipsychotics. However, cannabis use has been shown to increase the rate of relapse in patients with remitted FEP who both do and do not adhere to treatment. In the light of these and our findings, not only medication adherence, but also the type of antipsychotic medication might also affect relapse risk of psychotic disorders.^34^ Further, there are studies where treatment with clozapine^35,36^ and olanzapine^36^ have led to weaker craving for cannabis versus risperidone in people with schizophrenia, but the extant evidence base is too scarce to provide clinical recommendations.^37^ If replicated, our findings have clinical relevance to promote the use of drugs other than strong dopamine blockers for prevention of psychosis relapses after CIP.

Of the oral antipsychotic medications, clozapine was the most effective for preventing either psychosis or SUD relapses. Recent systematic review and meta-analysis suggests clozapine is superior to the other antipsychotic medications in people with schizophrenia and SUD and associated with significantly higher odds of remaining abstinent from substance use and decreased likelihood of psychiatric hospital admission.^38^ Cannabis use also associates with treatment resistance in schizophrenia^10^ where use of clozapine is indicated. The findings also align with our recent study, where clozapine was the most effective oral antipsychotic in relapse prevention for psychosis and SUD in FEP and a comorbid CUD sample.^20^

Almost three-quarters of the sample had used antipsychotics after their first episode of CIP. This is interesting, as there are no guidelines to how antipsychotic treatment should be prescribed after CIP. Olanzapine was the most frequently used antipsychotic medication and was effective in preventing relapses caused by both psychosis and SUD. Although it is considered as effective treatment for acute presentations of psychosis, it might not be feasible medication for long-term relapse prevention because of known metabolic and cardiovascular side-effects.^39^

Further, oral aripiprazole was the most effective non-clozapine antipsychotic in preventing psychosis relapses, but it did not reach statistical significance in relapse prevention for SUD. However, aripiprazole LAI was effective and the effect sizes were similar to other LAIs. This finding is likely related to non-adherence to oral medications and encourages the use of LAI formulations. Preliminary evidence and case reports suggest that partial agonists might be beneficial for the treatment of dual disorders where psychotic symptoms are present. This has been theorised to be partly related to their ability to bolster weakened prefrontal dopaminergic transmission that could improve cognitive dysfunctions and symptoms linked to lower dopaminergic functionality^40^ and also to reduce craving.^40,41^ However, further studies are required to prove whether aripiprazole and other partial agonists have a special role in treatment of dual disorders or SIP.

Similar to previous register studies^16,20,31^ antipsychotic polytherapy was effective in preventing psychosis or SUD relapses, suggesting that prescribers should consider it as a viable option for relapse prevention also after CIP. The combination of clozapine and aripiprazole is considered best in terms of relapse prevention,^42^ both of which are also associated with favourable substance use outcomes. In this relatively small sample, we did not study specific polytherapy combinations but that is an important topic for future studies.

Concerning somatic outcome, any antipsychotic use was a protective factor for subsequent hospital treatment caused by somatic disorder. However, none of the antipsychotics reached statistical significance for effectiveness, and hospital admission caused by somatic disorder was too rare to run medication modelling for most antipsychotics. Nonetheless, the use of these antipsychotics did not lead to increased severe physical morbidity leading to hospital admission. This is of importance, as antipsychotic use has been linked to adverse somatic outcomes.^39^

Our study has notable strengths, but also significant limitations. This study is the first to examine the long-term outcomes of different antipsychotic medications after CIP. Our novel findings contribute to the development of future clinical recommendations. The nationwide register-based data-set with information on all Swedish residents provides exceptional generalisability and the use of within-person analyses eliminates the effect of familial and genetic confounders.

This study identified individuals based on their first-time psychosis diagnosis, including those initially diagnosed with F12.5, and followed them over time. Not having information on the continuation of cannabis use should be seen as a limitation, as continued cannabis use associates with worse prognosis in psychosis than discontinued use.^43^ However, 69.7% of the sample were re-diagnosed with CUD (F12.x) by the end of the follow-up, suggesting persistence of these diagnoses after CIP. Because of power issues, we were not able to study whether there are specific antipsychotic medications that should be used as polytherapy and to run comprehensive medication modelling for somatic outcomes over even longer-term follow-up.

In particular, LAIs, clozapine and oral aripiprazole were associated with decreased risk of psychosis relapse after the first clinically diagnosed episode of CIP. This carries an important message that prescribers should consider more LAIs for better treatment outcomes after the first episode of CIP.

## Supporting information

Mustonen et al. supplementary material Mustonen et al. supplementary material

## Data Availability

The data used in this study cannot be made publicly available because of privacy regulations. According to the General Data Protection Regulation, the Swedish law SFS 2018:218, the Swedish Data Protection Act, the Swedish Ethical Review Act and the Public Access to Information and Secrecy Act, these types of sensitive data can only be made available for specific purposes, including research, that meet the criteria for access to this sort of sensitive and confidential data, as determined by a legal review. Readers may contact Professor Kristina Alexanderson (kristina.alexanderson@ki.se) regarding the data.

## Supplementary material

The supplementary material can be found at https://doi.org/10.1192/bjp.2025.72
