# Supplementary material for: Real-world effectiveness of antipsychotic medication in relapse prevention after cannabis-induced psychosis
Source: Br J Psychiatry. 2025 May 6;228(4):317–23. doi: 10.1192/bjp.2025.72 (PMC13051211; doi:10.1192/bjp.2025.72)
Supplement: Mustonen et al. supplementary material [file S0007125025000728sup001.docx]

**Online supplement table 1. Number of events, users, person-years and adjusted hazard ratios for psychosis hospitalization by specific medications.**

|  | Events | Users | Person-years | aHR (95%CI) |
| --- | --- | --- | --- | --- |
| **Antipsychotics** |  |  |  |  |
| No exposure | 1892 | 1754 | 10617,19 | Reference |
| Levomepromazine | 30 | 131 | 49,64 | 0.92 (0.59-1.44) |
| Perphenazine | NA | NA | NA | NA |
| Perphenazine LAI | 9 | 26 | 28,58 | 0.55 (0.25-1.22) |
| Haloperidol | 35 | 125 | 69,09 | 1.01 (0.66-1.54) |
| Haloperidol LAI | 14 | 22 | 18,37 | 1.14 (0.61-2.15) |
| Flupentixol | 5 | 22 | 20,42 | 0.88 (0.31-2.50) |
| Flupentixol LAI | NA | NA | NA | NA |
| Zuclopenthixol | 10 | 41 | 41,29 | 0.71 (0.32-1.61) |
| Zuclopenthixol LAI | 26 | 47 | 45,7 | 0.77 (0.47-1.26) |
| Clozapine | 28 | 54 | 119,25 | 0.56 (0.34-0.90) |
| Olanzapine | 404 | 1013 | 1031,96 | 0.82 (0.70-0.96) |
| Olanzapine LAI | 13 | 57 | 50,1 | 0.29 (0.16-0.55) |
| Quetiapine | 91 | 385 | 405,41 | 0.94 (0.69-1.27) |
| Risperidone | 72 | 261 | 210,27 | 0.91 (0.66-1.26) |
| Risperidone LAI | 18 | 40 | 45,46 | 0.55 (0.28-1.10) |
| Aripiprazole | 62 | 331 | 283,83 | 0.61 (0.43-0.88) |
| Aripiprazole LAI | 15 | 69 | 83,45 | 0.26 (0.14-0.49) |
| Paliperidone LAI 1M | 32 | 74 | 63,03 | 0.69 (0.45-1.08) |
| Paliperidone LAI 3M | 6 | 8 | 10,39 | 0.43 (0.09-2.03) |
| AP Polytherapy | 423 | 675 | 727,23 | 0.75 (0.64-0.89) |
| Cariprazine | 5 | 19 | 9,32 | 20.88 (1.99-218.64) |
| Paliperidone oral | 5 | 42 | 14,8 | 1.38 (0.48-3.95) |
| Other SG oral | NA | NA | NA | NA |
| Other FG oral | NA | NA | NA | NA |
| **ADHD medications** |  |  |  |  |
| No exposure | 3127 | 1767 | 13312,32 | Reference |
| Dexamfetamine | NA | NA | NA | NA |
| Methylphenidate | 30 | 206 | 307,89 | 0.67 (0.41-1.11) |
| Modafinil | NA | NA | NA | NA |
| Atomoxetine | 16 | 85 | 59,31 | 0.64 (0.32-1.26) |
| Lisdexamphetamine | 27 | 168 | 223,14 | 1.10 (0.61-1.98) |
| ADHD polytherapy | NA | NA | NA | NA |
| ***SUD medications*** |  |  |  |  |
| No exposure | 3145 | 1767 | 13749,18 | Reference |
| Disulfiram | 25 | 79 | 41,09 | 0.94 (0.48-1.82) |
| Acamprosate | NA | NA | NA | NA |
| Naltrexone | 10 | 60 | 36,73 | 1.39 (0.55-3.50) |
| Buprenorphine | 15 | 24 | 55,87 | 0.83 (0.27-2.56) |
| Metadone | 11 | 17 | 68,81 | 3.05 (0.80-11.69) |
| Multiple SUD drugs | NA | NA | NA | NA |
| **Antidepressants** |  |  |  |  |
| No exposure | 2704 | 1742 | 11565,26 | Reference |
| Clomipramine | 5 | 24 | 30,15 | 0.57 (0.15-2.12) |
| Amitriptyline | NA | NA | NA | NA |
| Nortriptyline | NA | NA | NA | NA |
| Fluoxetine | 22 | 118 | 164,43 | 0.75 (0.41-1.34) |
| Citalopram | 15 | 109 | 114,94 | 0.56 (0.29-1.10) |
| Paroxetine | 12 | 30 | 50,26 | 1.60 (0.67-3.77) |
| Sertraline | 104 | 447 | 538,89 | 0.75 (0.56-1.00) |
| Fluvoxamine | NA | NA | NA | NA |
| Escitalopram | 60 | 249 | 277,43 | 1.03 (0.71-1.49) |
| Moclobemide | NA | NA | NA | NA |
| Mianserin | NA | NA | NA | NA |
| Mirtazapine | 122 | 449 | 387,9 | 0.89 (0.69-1.15) |
| Bupropion | 13 | 155 | 86,75 | 0.94 (0.48-1.82) |
| Venlafaxine | 48 | 170 | 217,67 | 1.15 (0.75-1.76) |
| Reboxetine | NA | NA | NA | NA |
| Duloxetine | 25 | 91 | 101,98 | 1.30 (0.75-2.27) |
| Agomelatine | <5 | 20 | 9,38 | 5.28 (0.41-67.42) |
| Vortioxetine | 6 | 48 | 36,58 | 0.67 (0.26-1.73) |
| Rare antidepressants | NA | NA | NA | NA |
| Antidepressant polytherapy | 65 | 364 | 364,42 | 0.93 (0.62-1.39) |
| **Benzodiazepines and related drugs** | | | |  |
| No exposure | 2817 | 1755 | 12756,76 | Reference |
| Any benzodiazepine or related drug | 390 | 732 | 1231,3 | 1.19 (1.01-1.40) |
| **Mood stabilizers** |  |  |  |  |
| No exposure | 3020 | 1768 | 13280,77 | Reference |
| Carbamazepine | 11 | 41 | 42,23 | 0.93 (0.44-1.99) |
| Valproic acid | 89 | 168 | 206,7 | 0.93 (0.70-1.25) |
| Lamotrigine | 15 | 115 | 159,81 | 0.68 (0.34-1.37) |
| Topiramate | NA | NA | NA | NA |
| Lithium | 60 | 107 | 217,85 | 0.98 (0.67-1.43) |
| Mood stabilizer polytherapy | 8 | 66 | 64,51 | 0.46 (0.20-1.07) |

**Methods (Sensitivity Analysis)**

As only patients with variation in the exposure and outcome directly contribute to a within-individual analysis, we conducted between-individual Cox-regression analyses as sensitivity analyses for the main outcome. In these analyses all individuals diagnosed with cannabis-induced psychosis contribute directly to the estimates, irrespective whether they had used antipsychotics or relapsed during the follow-up.

Between-individual models were adjusted for the temporal order of antipsychotic medication treatments and the time-varying use of other psychotropic medications. These medications were categorized based on their ATC codes as medications for SUDs (N07BB, N07BC), medications for attention-deficit/hyperactivity disorder (N06BA), mood stabilizers (N03AF01, N03AG01, N03AX09, N05AN01), antidepressants (N06A), benzodiazepines and related drugs (N05BA, N05CD, N05CF). These models were also adjusted for age, sex, granted disability pension, number of previous hospitalizations due to cannabis use disorder, and events of brain injury, epilepsy, personality disorders, accidental overdoses, and suicide attempts. Covariate definitions are presented in Supplement Table 2.

**Results (Sensitivity Analysis)**

In between-individual analyses, aripiprazole LAI (aHR 0.54; 95% CI 0.30-0.96) was associated with a reduced risk of psychosis hospitalization, while antipsychotic polytherapy increased the risk of psychosis hospitalization (aHR 1.30; 95% CI 1.10-1.55). The other antipsychotics did not reach statistical significance: olanzapine LAI (aHR 0.58; 95% CI 0.31-1.01), paliperidone LAI (aHR 0.77; 95% CI 0.47-1.26), risperidone LAI (aHR 0.83; 95% CI 0.49-1.40), oral aripiprazole (aHR 0.87; 95% CI 0.68-1.21), clozapine (aHR 1.00; 95% CI 0.55-1.82), quetiapine (aHR 1.04; 95% CI 0.83-1.32), oral risperidone (aHR 1.05; 95% CI 0.78-1.41), oral olanzapine (aHR 1.08; 95% CI 0.94-1.24), FG-LAIs (aHR 1.20; 95% CI 0.78-1.85), and other oral antipsychotic monotherapy (aHR 1.23; 95% CI 0.97-1.56). The effect sizes were generally smaller than in within-individual models but ranked similarly to primary models.

**Online supplement table 2. Covariate definitions.**

| Covariate | Definition | Register source | Models utilizing the covariate |
| --- | --- | --- | --- |
| Order of treatments | Temporal order of treatments, continuously updated in the models and categorized as first, second, third and >third | PDR | W, B |
| Time since cohort entry | Continuously updated in the models, categorized as 0-1, 1-3 and >3 years |  | W |
| Antidepressants | Continuously updated in the models, ATC N06A | PDR | W, B |
| Benzodiazepines and related drugs | Continuously updated in the models, ATC N05BA, N05CD, N05CF | PDR | W, B |
| ADHD medications | Continuously updated in the models , ATC N06BA | PDR | W, B |
| SUD medications | Continuously updated in the models, ATC N07BB medications used in alcohol dependence, N07BC medications used in opioid use disorder | PDR | W, B |
| Age | Age at cohort entry | LISA | B |
| Gender | Man vs. woman | LISA | B |
| Disability pension | Continuously updated in the models, no vs. yes after the first granted decision. | MiDAS | B |
| Number of previous hospitalizations due to cannabis use disorder | Continuously updated in the models and categorized as 0, 1, 2-3 vs. >3 | NPR | B |
| ADHD | Continuously updated in the models with status ”no” until the first diagnosis ICD-10 F90 occurred and ”yes” thereafter. | NPR | B |
| Personality disorders | Continuously updated in the models with status ”no” until the first diagnosis ICD-10 F60-F69 occurred and ”yes” thereafter. | NPR | B |
| Epilepsy | Continuously updated in the models with status ”no” until the first diagnosis ICD-10 G40 occurred and ”yes” thereafter. | NPR | B |
| Brain injury | Continuously updated in the models with status ”no” until the first diagnosis ICD-10 S06 occurred and ”yes” thereafter. | NPR | B |
| Previous suicide attempt | Continuously updated in the models with status ”no” until the first diagnosis ICD-10 X60-X84, Y10-Y34 occurred and ”yes” thereafter. | NPR | B |
| Previous accidental overdose | Continuously updated in the models with status ”no” until the first diagnosis ICD-10 X40-X49 occurred and ”yes” thereafter. | NPR | B |
| PDR: Prescribed Drug Register, LISA: The Longitudinal Integration Database for Health Insurance and Labor Market Studies, NPR: National Patient register, MiDAS: the Microdata for Analyses of Social Insurance.  W=within-individual models, B=between-individual models. | | | |
